# 2208. Rates of Medically-Attended RSV among US Adults: A Systematic Review and Meta-Analysis

**DOI:** 10.1093/ofid/ofac492.1827

**Published:** 2022-12-15

**Authors:** John M McLaughlin, Farid L Khan, Elizabeth Begier, David Swerdlow, Luis Jodar, Ann R Falsey

**Affiliations:** Pfizer, Collegeville, Pennsylvania; Pfizer, Collegeville, Pennsylvania; Pfizer Vaccines, Dublin, Dublin, Ireland; Pfizer, Collegeville, Pennsylvania; Pfizer Vaccines, Dublin, Dublin, Ireland; University of Rochester School of Medicine and Dentistry, Rochester, NY; Rochester Regional Health, Rochester, NY, USA, Rochester, New York

## Abstract

**Background:**

Adult respiratory syncytial virus (RSV) vaccines are in late stages of development. A comprehensive synthesis of adult RSV burden is needed to inform public health decision-making.

**Methods:**

We performed a systematic review and meta-analysis of studies describing the incidence of medically-attended RSV (MA-RSV) among US adults. We also identified studies reporting nasopharyngeal (NP) or nasal swab RT-PCR results with paired serology (four-fold-rise) or sputum (RT-PCR) to calculate RSV detection ratios quantifying improved diagnostic yield after adding a second specimen type (*ie*, serology or sputum).

**Results:**

We identified 14 studies with 15 unique MA-RSV incidence estimates, all based on NP or nasal swab RT-PCR testing alone. Pooled annual RSV-associated incidence per 100,000 adults ≥65 years of age was 178 (95%CI: 152‒204; n=8 estimates) hospitalizations (4 prospective studies: 189; 4 model-based studies: 157), 133 (95%CI: 0‒319, n=2) emergency department (ED) admissions, and 1519 (95%CI: 1109‒1929, n=3) outpatient visits. Based on 6 studies, RSV detection was ∼1.5 times higher when adding paired serology or sputum. After adjustment for this increased yield, annual RSV-associated rates per 100,000 adults ≥65 years were 267 hospitalizations (UI: 228‒306) (prospective: 282; model-based: 236), 200 ED admissions (UI: 0‒478), and 2278 outpatient visits (UI: 1663‒2893). Persons < 65 years with chronic medical conditions were 1.2−28 times more likely to be hospitalized for RSV depending on risk condition.

**Conclusion:**

The true burden of RSV has been underestimated and is significant among older adults and individuals with chronic medical conditions. A highly effective adult RSV vaccine would have substantial public-health impact.

**Disclosures:**

**John M. McLaughlin, PhD**, Pfizer: Employee|Pfizer: Stocks/Bonds **Farid L. Khan, MPH**, Pfizer: Employee|Pfizer: Stocks/Bonds **Elizabeth Begier, M.D., M.P.H.**, Pfizer: Employee|Pfizer: Stocks/Bonds **David Swerdlow, MD**, Pfizer: Advisor/Consultant|Pfizer: Stocks/Bonds **Luis Jodar, PhD**, Pfizer: Employee|Pfizer: Stocks/Bonds **Ann R. Falsey, MD**, BioFire Diagnostics: Grant/Research Support|Janssen: Grant/Research Support|Merck, Sharp and Dohme: Grant/Research Support|Novavax: Advisor/Consultant|Pfizer: Grant/Research Support.

